# Implantable Light‐Powered Human Designer Cells for Electrical Energy Generation

**DOI:** 10.1002/adma.202502618

**Published:** 2025-09-03

**Authors:** Shuai Xue, Zhihua Lin, Debasis Maity, Preetam Guha Ray, Mingqi Xie, Martin Fussenegger

**Affiliations:** ^1^ Department of Biosystems Science and Engineering ETH Zürich Klingelbergstrasse 48 Basel CH‐4056 Switzerland; ^2^ Westlake Laboratory of Life Sciences and Biomedicine Hangzhou Zhejiang 310024 China; ^3^ Faculty of Science University of Basel Klingelbergstrasse 48 Basel CH‐4056 Switzerland

**Keywords:** electrogenetics, genetic engineering, light‐sensitive membrane proteins, photovoltage, solar cells, synthetic biology

## Abstract

Herein, an implantable, miniature biohybrid device has been developed that utilizes light‐dependent ion‐gradient formation by genetically engineered human designer cells, expressing light‐activated ion channels and proton pumps to generate electrical potential and deliver electrical energy. These designer cells are cultured in custom‐designed polycarbonate chambers, connected by electrodes and separated from an ion reservoir by a proton‐selective Nafion membrane. Upon illumination, the light‐activated channels and pumps on the designer cells establish a sustained proton gradient across the Nafion membrane, which drives an electrical current in the external circuit. When exposed to simulated ambient sunlight of 3 mW cm^−^^2^ of intensity, a single solar collection device (SCD), containing these designer cells, appropriately sized for subcutaneous implantation in mice, generates an electrical potential of ≈0.4 V. By connecting multiple SCDs in series and increasing the cell suspension volume, the output can be scaled up sufficiently to power a commercial light‐emitting diode. Thus, this study demonstrates the feasibility of a photovoltaic system based on optogenetically engineered mammalian cells for powering bioelectronic implants or wearable devices.

## Graphical abstract

Inspired by the eel's electric organ, a miniature implantable biohybrid device is developed to generate electricity under light activation. The device utilizes a light‐dependent ion gradient formation that induced by genetically engineered human designer cells that express light‐sensitive membrane ion channels and proton pumps, working in synergy with the device to generate electrical potential and deliver electrical energy.

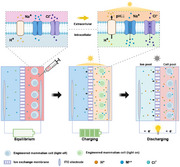

## 1 Introduction

Functionally linking biological entities with electronic devices relies on interfacial designs that enable bidirectional transfer of electricity.^[^ ^1^, ^2^ ^]^ Advances in electrogenetics have led to the construction of a variety of molecular interfaces that can program cellular genetics in response to electrical stimuli, through energy transfer from electronic to biological components.^[^ ^3^, ^4^, ^5^, ^6^, ^7^ ^]^ Conversely, for energy conversion from biological to electronic devices, several cell‐based bio‐electrochemical systems have been engineered in plants,^[^ ^8^, ^9^, ^10^, ^11^ ^]^ bacteria,^[^ ^12^, ^13^ ^]^ and photosynthetic microorganisms.^[^ ^14^, ^15^, ^16^, ^17^ ^]^ Particularly, mammalian retinal cells have been elegantly modified to serve as potential electron donors by secreting nicotinamide adenine dinucleotide phosphate (NADPH) onto an anode, generating a current density in µA/cm^2^ range.^[^ ^18^ ^]^ However, photosynthetic bio‐harvesting systems rely on electron‐transfer chains, which generate high concentrations of superoxide ions or cause excessive heating; these are unsuitable for in vivo applications, as they are incompatible with the physiological environment of mammalian cells.^[^ ^19^, ^20^ ^]^ Additionally, approaches based on cell‐membrane‐spanning electron transport chains typically require the application of an external electrical potential to initiate the system, and as is the case with capacitors in general, the amount of harvested electrical energy is relatively small and instantaneously discharged.^[^ ^20^, ^21^ ^]^ Thus, effective strategies for harnessing mammalian cells to generate electrical potential that can be stored in the form of chemical energy and converted to electrical energy on demand, which is a phenomenon characteristic of batteries, have remained elusive.

The electric organ of an electric eel utilizes asymmetrically distributed ion channels and ion pumps on individual cell membranes to establish ion gradients across the cells; this robustly translates changes in cation concentrations into voltage production to harvest electricity from ionic imbalances.^[^ ^21^ ^]^ Inspired by this approach, various abiotic systems, employing analogous principles, have been constructed to generate electricity.^[^ ^22^, ^23^, ^24^, ^25^ ^]^ Furthermore, solar energy can be considered as a continuous power source, supported by advances in the application of various light‐sensitive ion exchange systems for bioengineering. Examples include the blue light‐dependent rhodopsin channel ChR2(H134R), green light‐responsive chimeric opsin channel C1V1(t/t),^[^ ^26^ ^]^ orange‐light‐dependent halorhodopsin pump eNpHR3.0, derived from *Natromonas pharaonic*,^[^ ^27^ ^]^ blue light‐dependent channel iChloC,^[^ ^28^ ^]^ and optimized yellow/green‐light‐activated outward proton pumps, such as eArch3.0 and eMac3.0, originally derived from *Halorubrum sodomense* and *Leptosphaeria maculans*, respectively.^[^ ^29^ ^]^

Here, to construct a mammalian‐cell‐based photovoltaic system, we adopted a synthetic biology‐inspired approach to construct human cells with transgenic expression of light‐sensitive membrane proteins aimed at inducing either inward ion fluxes of cations (M^n+^, representing physiological metal ions, such as Na^+^, K^+^, Ca^2+^) or chloride ions (Cl^−^) and/or outward ion fluxes of protons (H^+^). Upon exposure to light, these channels mediate rapid changes in extracellular ion concentrations within physiological and non‐cytotoxic ranges. We considered that such light‐induced shifts in ion distributions could be exploited to create a sustained concentration gradient across two fluid chambers, separated by an ion‐exchange membrane; this would result in a unidirectional ion flow capable of driving electrical outputs, analogous to an electric eel's organ. First, we investigated a range of light‐sensitive proteins, expressed alone and in various combinations, to optimize induced ionic strength and dynamics. We designated these optimized stably transgenic human designer cells as mammalian “solar cells” (SCs). Next, to harness solar energy to autonomously power and control electronic devices, we built a custom‐designed cultivation system for the SCs, called the “solar collection device” (SCD). Finally, we demonstrated that light‐(re)chargeable electricity production using implantable SCDs could provide sufficient power to drive a conventional light‐emitting diode (LED). We believe this light‐harvesting design has the potential to power optogenetic, light‐triggered electrogenetic devices, as well as wearable sensors,^[^ ^30^ ^]^ providing an alternative approach to stimulate the electrogenetic.^[^ ^5^, ^6^ ^]^

## 2 Results

### 2.1 Engineering and Validation of Photovoltaic Human Designer Cells

**Figure 1:**
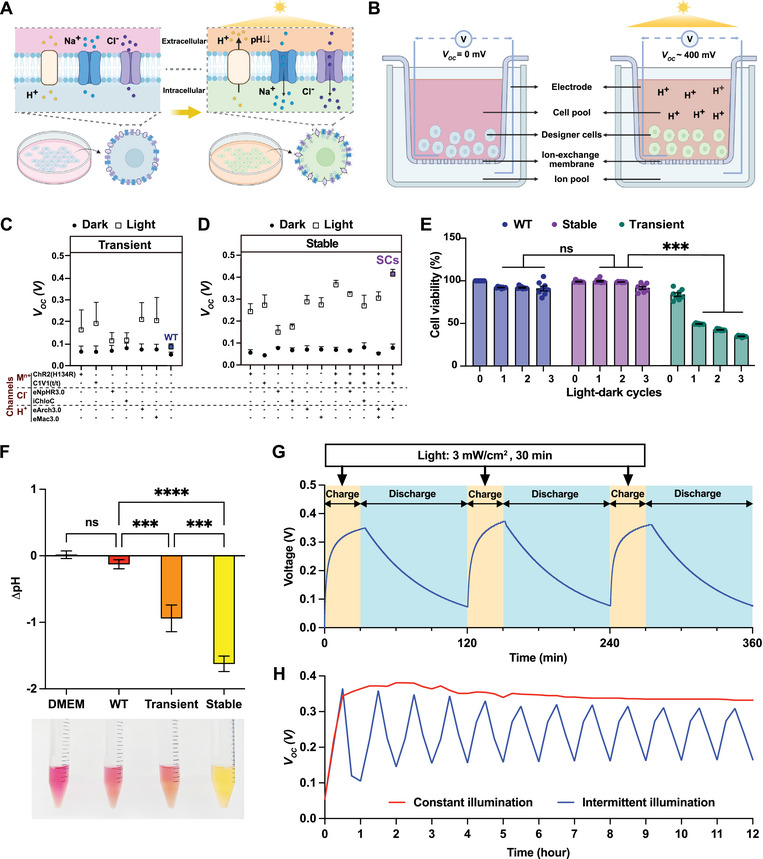
Conceptual design of a mammalian cellular photovoltaic system. A) Schematic representation of photosensitive designer cells, created in BioRender. Lin, Z. (2025) https://BioRender.com/534ywqf. B) Scheme of SCD1 with Transwell insert for photovoltage generation, created in BioRender. Lin, Z. (2025) https://BioRender.com/534ywqf. C) Voltage generation by SCD1 with (C) transiently and D) stably transfected designer HEK‐293 cells engineered to express different photosensitive membrane proteins (cell density, 1 × 10^7^ cells mL^−1^; light, 3 mW cm^−2^, 30 min). The voltage between the cell pool (0.5 mL) and the ion pool (0.2 mL) was measured immediately after illumination. E) Cell viability evaluation of wild‐type cells (WT), and designer cells stably and transiently expressing hChR2(H134R), C1V1(t/t), and eArch3.0 during multiple light‐dark cycles (3 mW cm^−2^ for 30 min, dark for 1 h). F) Light‐dependent pH change of cell supernatant (cell density, 1 × 10^7^ cells mL^−1^; white light intensity, 3 mW cm^−2^, 30 min). (F) Illumination‐dependent pH change. Culture supernatants from cell pools containing 1 × 10^7^ Cells mL^−1^ or parental WT cells were profiled after a 30 min charge step at 3 mW cm^−2^. ΔpH represents the pH difference between the cell pool and the ion pool. G) Charge‐discharge curve of monoclonal cells stably expressing hChR2(H134R), C1V1(t/t) and eArch3.0 (cell density, 1 × 10^7^ cells mL^−1^; white light intensity, 3 mW cm^−2^, 30 min; resistor device, 10 MΩ). H) Reversible voltage generation in 12 h with constant or intermittent illumination (white light, 3 mW cm^−2^, resistor device, 2 MΩ). All data have been presented as mean ± SEM, data in (C, D, F, G, H) *n* = 3, (E) *n* = 8. Statistical significance was analyzed by two‐way ANOVA with Tukey's test (ns *p* ≥ 0.05; ^**^ *p* < 0.01; ^***^ *p* < 0.001; ^****^ *p* < 0.0001).

To generate an electrical potential from stable ion concentration gradients, precise control of ion distribution in a specific aqueous solution is a prerequisite.^[^ ^23^ ^]^ The use of mammalian cells engineered for transgenic expression of light‐sensitive ion channels or pumps is expected to promote light‐inducible ion traffic between the intracellular and extracellular milieus, thereby inducing rapid and reversible changes in the ion potential or pH in the culture supernatant (**Figure** **1**A). To develop designer cells capable of such light‐activated regulation of electrogenic ion gradients, we first transiently transfected human HEK‐293 cells, a widely used cell model system, with various representative light‐sensitive ion channels or pumps; specifically, we focused on two metal cation (M^n+^) channels hChR2(H134R)^[^ ^26^ ^]^ and C1V1(t/t),^[^ ^26^ ^]^ the chloride ion (Cl^‐^) pump eNpHR3.0,^[^ ^27^ ^]^ the chloride ion channel iChloC,^[^ ^28^ ^]^ and two optimized proton (H^+^) pumps eArch3.0 and eMac3.0.^[^ ^29^ ^]^ Engineered cells expressing various combinations of these proteins were characterized, as described below, to identify the optimal combination.

To exploit light‐dependent ion concentration differences generated by these cells to produce electricity, we first designed an initial version of a prototype insert‐based SCD (designated as SCD1) (Figure 1B). This device consists of a cell culture insert with a Nafion coating as an ion‐exchange membrane docked to a tissue culture plate, together with two platinum electrodes —one positioned inside the insert used to grow the mammalian designer cells (called the cell pool), and the other placed in a cell‐free tissue culture system at the bottom (called the ion pool). At equilibrium, the cell and the ion pools have similar initial ion concentrations. Because the Nafion coating selectively allows cation exchange, exposure to light rapidly increases the cation concentration in the cell pool, establishing a sustained ion gradient across the proton‐exchange membrane and generating an ionic potential that ultimately generates an electrical potential in the circuit (Figure 1B). The cells were then tested for open‐circuit voltage (*V_OC_*) generation when seeded (5 × 10^6^ cells) in the cell pool of the insert‐based SCD1. A custom‐designed broad‐spectrum white light (420–720 nm, 3 mW cm^−2^) was used to simulate the power density of sunlight, while taking into account the different responsive wavelength ranges of the proteins we studied.

In contrast to the wild‐type (WT) HEK‐293 cells, we observed an increased *V_OC_* upon illumination of designer cells transiently transfected with single channels (Figure 1C). A significantly higher voltage was induced by the transport of metal cations (hChR2(H134R) or C1V1(t/t)) and protons (eArch3.0 and/or eMac3.0) than by that of chloride ions (iChloC or eNpHR3.0). This finding was consistent with previously reported hyperpolarization experiments, suggesting intrinsic differences in ion transfer efficiency.^[^ ^29^, ^31^ ^]^ To maximize the induced voltage and increase the tolerance of the cells to ionic changes over a prolonged working time, we selected stably transgenic monoclonal cells expressing the photosensitive ion channels (**Table** **1**) that showed the highest fluorescence from co‐expressed fluorophores (Table S1, Supporting Information). These stable cells exhibited higher levels of light‐stimulated voltage generation than their transiently transfected counterparts (Figure 1D). To harness solar energy over a broad wavelength range, we co‐expressed combinations of these photosensitive elements, resulting in a variety of optimized voltage outputs from the co‐expressed variants (Figure 1D). A representative increase in voltage output was obtained by co‐expressing cation channels of the same type with different peak excitation wavelengths ((hChR2(H134R): ≈460 nm; C1V1(t/t): 550–560 nm).^[^ ^32^ ^]^ Other stable cell lines were engineered based on this dual‐cation‐channel setup, including the addition of chloride ion channels or proton pumps. The highest voltage output from SCD1 (0.408 V) was provided by stable cells co‐expressing hChR2(H134R), C1V1(t/t), and eArch3.0; these cells are referred to as SCs hereafter. An anion exchange membrane (AEM)‐based SCD1 was also constructed to test chloride‐based stably transfected cells, and comparable voltage output to that obtained with the Nafion membrane was produced (Figure S1A, Supporting Information). The chloride ion concentration dropped from ≈133 to 118.9 mM and 91.4 mM for engineered cells stably expressing eNpHR3.0 and iChloC after light illumination (3 mM cm^−2^, 30 min, Figure S1B, Supporting Information). However, considering the low biocompatibility of AEM (Figure S1C, Supporting Information), all subsequent experiments were performed with Nafion.

**Table 1:** Light‐inducible ion channels and proton pumps were used in this study. For resistance selection, puromycin (Puro), blasticidin (Blast), and zeocin (Zeo) were applied at concentrations of 1, 5, and 100 µg mL^−1^, respectively. For ion transfer, M^n+^, H^+^, and Cl^−^ refer to cations, protons, and chloride ions, respectively.

Stable cell no.	Transgene(s)	Resistance	Ion transfer	Responsive wavelength (nm)
613	PsChR2	Blast	M^n+^ (inward)	green (510 nm)
630	hChR2(H134R)	Puro	M^n+^ (inward)	blue (460 nm)
631	C1V1(t/t)	Blast	M^n+^ (inward)	green (550 nm)
720	eArch3.0	Zeo	H^+^ (outward)	yellow (566 nm)
721	eMac3.0	Zeo	H^+^ (outward)	green (540 nm)
632	eNpHR3.0	Zeo	Cl^−^ (inward)	orange (589 nm)
633	iChloC	Zeo	Cl^−^ (inward)	blue (465 nm)
610	iChR2^++^	Puro	Cl^−^ (inward)	blue (465 nm)
3031	hChR2(H134R), C1V1(t/t)	Puro, Blast,	M^n+^ (inward)	white
3012	hChR2(H134R), C1V1(t/t), eNpHR3.0	Puro, Blast, Zeo	M^n+^ (inward), Cl^−^ (inward)	white
3013	hChR2(H134R), C1V1(t/t), iChloC	Puro, Blast, Zeo	M^n+^ (inward), Cl^−^ (inward)	white
3031.01	hChR2(H134R), C1V1(t/t), eMac3.0, eArch3.0	Puro, Blast, Zeo	M^n+^ (inward), H^+^ (outward)	white
3031.20 (SC)	hChR2(H134R), C1V1(t/t), eArch3.0	Puro, Blast, Zeo	M^n+^ (inward), H^+^ (outward)	white

The stable SCs not only produced the highest *V_OC_* (Figure 1D), but also maintained high viability compared with their transiently transfected counterparts upon repeated cycles of light exposure (3 mM cm^−2^, 30 min) (Figure 1E). Note that the enhanced cell viability is due to the well‐known phenomenon of environmental adaption during the screening process.^[^ ^33^ ^]^ The extracellular proton level increased after a single 30‐min exposure, as indicated by a decrease in the pH of the cell pool. The resulting pH difference between the ion pool and cell pool is significantly higher for stably transfected cells (−1.62 ± 0.11) compared to transiently transfected cells (−0.94 ± 0.20) or wild‐type (WT) parental cells (−0.12 ± 0.07) in accordance with the visible color change of the pH indicator (phenol red) in the Dulbecco's modified Eagle's medium (DMEM) culture medium (Figure 1F). Despite the systematic pH drop in the WT control group, which arises from cell metabolic activities, the pH decrease from 7.4 to 5.8 represents an almost 40‐fold increase in proton concentration. To test the electrical performance of the design, a charge–discharge cycle was started with a 30‐min “charge” step (light on) to induce an ion concentration difference between the cell and ion pools for *V_OC_* generation, followed by a 90‐min “discharge” step in the dark with an external resistor device (10 MΩ) in the circuit (Figure 1G). Over three test cycles, the SC‐loaded insert‐based SCD1 consistently and reversibly produced *V_OC_* upon exposure to white light, and the electrical energy, thus produced, was then consumed by the connected resistive device, leading to a decrease in *V_OC_*. Notably, with a resistor of 2 MΩ, *V_OC_* was strictly dependent on the intermittent light exposure, while the device generated a stable output potential when exposed to continuous illumination (Figure 1H).

### 2.2 Design and Validation of an Implantable Device, SCD2

**Figure 2:**
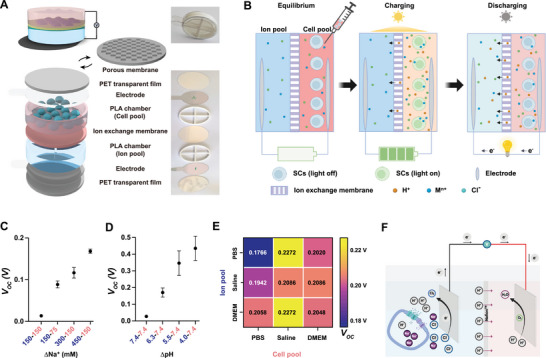
Development of the implantable device SCD2. A) Schematic representation of chamber‐based SCD2. B) Schematic representation of the mechanism of the SCD2, created in BioRender. Lin, Z. (2025) https://BioRender.com/534ywqf. C,D) Voltage generation of SCD2 with different (C) NaCl concentration or (D) pH in the cell pool (pink) or ion pool (blue) immediately after solution loading. E) Voltage generation of SCD2 with different physiological media (PBS, saline solution, or native DMEM) in the ion or cell pools. (cell density, 1 × 10^7^ cells mL^−1^; light intensity, 3 mW cm^−2^, 15 min). F) Electrochemical mechanism for electricity generation, created in BioRender. Maity, D. (2025) https://BioRender.com/ru96xb4. The movement of H⁺ ions across the Nafion^TM^ membrane can be described by the process “H⁺_intracellular → H⁺_extracellular → H⁺_through Nafion”. When a small resistive device is present, Cl^−^ ions are oxidized at the anode to produce free electrons (cell pool, 2Cl^−^ → Cl_2_ + 2e^−^), while charge neutrality is achieved at the cathode by reduction of oxygen to water (ion pool, O_2_ + 4H^+^ + 4e^−^→ 2H_2_O). Data in (C, D) have been presented as mean ± SEM and *n* = 3.

To design a device that allows the implementation of SCs in the form of a portable or implantable “power bank” that would be safe and compatible with everyday use, a number of requirements must be met. First, the device should be sealed and waterproof to prevent cell leakage or contamination. Second, the device should ideally have an appropriate geometry for implantation (flat or rounded).^[^ ^24^ ^]^ Third, the sealed device must allow sufficient oxygen and nutrients to be supplied to the SCs in the cell pool to maintain their viability. Satisfying these requirements, we developed a second‐generation miniaturized SCD, which we designated as SCD2, for implantation in mice. SCD2 consists of a commercially available ion‐exchange membrane (Nafion 117) sandwiched between two 3D‐printed polylactic acid (PLA) scaffolds, separating the cell‐and ion‐pool chambers, each of which contains a biocompatible conductive electrode. The device is covered with waterproof polyethylene terephthalate (PET) films for in vitro experiments (**Figure** **2**A). For in vivo applications, a biocompatible porous polycarbonate membrane can replace the top PET membrane of the cell pool to enable nutrient exchange with the host vasculature. Living SCs are then injected into the cell pool of SCD2 and counter solution into the ion pool (Figure 2B). The use of transparent materials for the major surfaces of SCD2, including the PET or polycarbonate membrane cover, conductive electrodes, and Nafion membrane, should ensure good light penetration and minimize power loss.

To optimize the functionality of the prototype SCD2, we first simulated the cation changes in both the cell and ion pools to calibrate the *V_OC_* produced with different saline (NaCl) concentrations (75–150 and 150–450 mM in the cell and ion pools, respectively; Figure 2C). The simulated *V_OC_* production increased as the Na^+^ concentration difference across the Nafion membrane increased. We then maintained the pH at 7.4 in the cell pool and mimicked typical physiological pH levels (4.0, 5.5, 6.3, and 7.4) in an NaCl solution (150 mM) in the ion pool. This revealed a similar dependence of *V_OC_* on the proton concentration difference (Figure 2D). Note that the voltage output was non‐linear (Figure 2C,D) due to the diffusion coefficient loss of 10‐ to 20‐fold across the Nafion membrane compared with that in the bulk solution.^[^ ^34^ ^]^ The ion pool was then filled with phosphate‐buffered saline (PBS), saline or cell culture medium, and cells were grown in the same medium in the cell pool (Figure 2E). Upon illumination, the generated voltage was higher when the ion pool was loaded with saline or cell culture medium, in contrast to the case of loading with PBS, which buffered the proton changes and showed the lowest light‐dependent voltage generation (Figure 2E). These results further established that voltage generation was critically dependent on the proton gradient, as expected (Figure 1).

Following the preliminary simulation and evaluation of SCD2, we hypothesized that current generation by the proton pumps might involve a series of finely tuned electrochemical processes (Figure 2F). Upon light illumination, the cation channel opens, and cation influx induces proton efflux due to cell membrane depolarization; then the protons are pumped out of the cell by the proton pump. Consequently, the pH in the extracellular region of the cell pool decreases. The movement of protons across the Nafion membrane from the proton‐rich cell pool to the proton‐poor ion pool maintains the charge equilibrium and induces electron transfer in the outer electric circuit. As a non‐Faradaic cell, the initial potential would not induce any significant electrochemical reactions. Nevertheless, if the electrode potential exceeds the chloride oxidation potential, as the Cl^−^ ions travel to the anode in the cell pool chamber, the risk of toxic chlorine gas being generated together with free electrons would surface; this gas would flow through the external circuit to the cathode, where charge neutrality would be restored when water molecules are formed from the released protons (H⁺), electrons (e^−^), and oxygen. However, this could only happen if the current density exceeds 1 mA cm^−2^,^[^ ^35^ ^]^ which is significantly higher than the levels reached in our experiments.

### 2.3 Electrical Characterization of SCD2 and Validation in Mice

**Figure 3:**
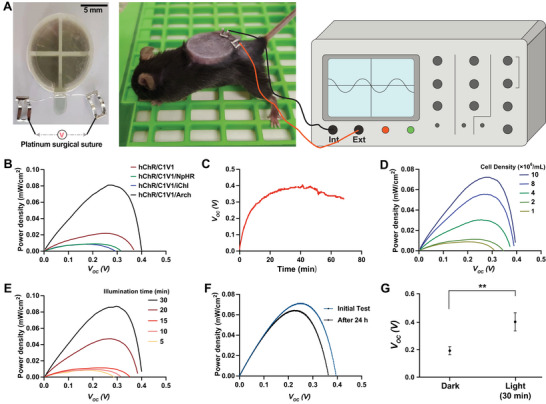
In vivo electrical characterization of subcutaneously implanted SCD2 in mice. A) Images of ex vivo SCD2 connected with platinum surgical sutures for implantation and mice subcutaneously implanted with SCD2 for electrical characterization. The sutures penetrated the mouse skin to the electrodes, and the silver wires connected the sutures and the ex vivo electrical detection device. B) Output power density of stable SCs with different photosensitive membrane proteins in SCD2. C) Time‐dependent output voltage of SCD2. D) SC‐density‐dependent output power density and voltage (light intensity, 3 mW cm^−2^, 30 min). E) Illumination‐time‐dependent output power density and voltage (cell density, 1 × 10^7^ cells mL^−1^; light intensity, 3 mW cm^−2^). F) Output power density of SCD2 before and after 24 h of dark treatment. G) Output voltage of subcutaneously implanted SCD2. Experimental parameters were kept constant in (B, C, F, and G) (cell density, 1 × 10^7^ cells mL^−1^; light intensity, 3 mW cm^−2^, 30 min). All data in (B–G) have been presented as mean ± SEM and *n* = 3 independent experiments. Statistical significance was analyzed by one‐way ANOVA with Dunnett's multiple comparisons test (ns *p* > 0.05; ^**^ *p* < 0.01).

Next, we embedded the SCD2 devices subcutaneously in mice for electrochemical assessment. To quantify the photovoltaic performance in vivo, a pair of platinum surgical sutures was employed for the collection of the generated electricity from the embedded SCD2 (**Figure** **3**A). The light illumination was maintained at 3 mW cm^−2^ by adjusting the distance from the lamp to the skin of the mouse. The SCs produced the highest power density (0.08 µW cm^−2^) among various cells stably expressing combinations of photosensitive elements, hChR2/C1V1, hChR2/C1V1/NpHR, hChR2/C1V1/iChloc, and hChR2/C1V1/eArch3.0 (SCs) in SCD2 under light illumination (Figure 3B); this was consistent with the voltage measurements shown in Figure 1C. Therefore, we continued with the SCs for the following in vivo experiments. A 30–40 min exposure to light illumination was sufficient to charge an SCD2 containing 1 x 10^7^ cells per mL; at this time point, the output voltage reached saturation, with an output power of ≈0.1 µW (Figure 3C). It should be noted that the porous membrane in SCD2 facilitates the exchange of small molecules between the encapsulated cell pool and the host system. Consequently, pH differences across the Nafion membrane are gradually balanced by the host's metabolic regulation, thereby reducing the pH gradient across the membrane, which results in a drop in the output voltage. For practical applications, this mild instability could be compensated by integrating capacitors or additional stabilisation electronics to ensure consistent power output. The total power density of the SCD2, measured in the steady‐state under constant illumination after 30 min of continuous exposure, increased with increasing cell density in the cell pool from 1 × 10^6^ to 1 × 10^7^ cells per mL (Figure 3D), and with increasing illumination time from 5 to 30 min (Figure 3E); however, both peaked at ≈0.08 µW cm^−2^, indicating that the maximum power‐generating capacity of the SCD2 had been reached (with an photo‐electric conversion efficiency of ≈0.0027%). At 24 h after the initial 30‐min illumination, the device generated a comparable power density (Figure 3F) and voltage output (Figure 3G) in response to a second 30‐min illumination, confirming the reversibility of the photovoltage output from the implant in mice. This, in turn, suggested the preservation of cell functionality and systemic integrity. The experimental mice also showed no apparent behavioral changes, further indicating that the illumination for powering SCD2 in vivo had no adverse effects.

### 2.4 Modularity and Scalability of SC‐Based Devices

**Figure 4:**
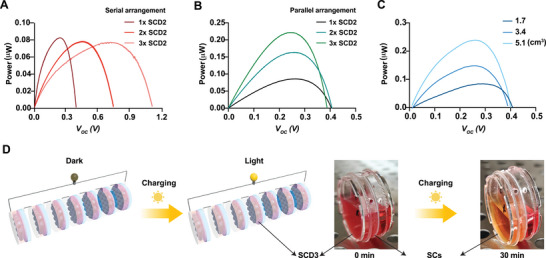
Scalability of SCDs. A,B) Device‐amount‐dependent output power density and voltage of SCD2 arrays connected (A) in series and (B) in parallel. C) Cell‐suspension‐dependent output power density of SCD2 (cell density, 1 × 10^7^ cells mL^−1^; light intensity, 3 mW cm^−2^, 30 min). D) Schematic illustration and pictures of serially connected SCD3 powering an LED after charging (cell density, 2 × 10^7^ cells mL^−1^; light intensity, 3 mW cm^−2^, 30 min; LED input threshold, 2.3 V; Movie S1, Supporting Information). Data in (A, B, C) have been presented as mean ± SEM and *n* = 3.

To mimic the operational functionality of power sources for electronic devices in everyday life, we connected single SCD2 units in different configurations. Connecting three SCD2 units in series resulted in a threefold increase of the maximum voltage to 1.2 V, compared with that of a single SCD2 at 0.4 V (**Figure** **4**A), while parallel placement of the three devices resulted in a threefold increase in the maximum output power level to 0.24 µW from that of a single SCD at 0.08 µW (Figure 4B). Furthermore, the power output increased linearly with increasing volume of cell suspension under the same simulated sunlight at a power density of 3 µW cm^−2^ (Figure 4C), indicating that the power output is tunable simply by making dimensional changes in a single device with a fixed cell density. Next, as a proof‐of‐concept for a scaled‐up device as a realistic power source for electronic devices, we designed a third‐generation device, which we designated as SCD3, with a much larger working area of ≈9.62 cm^2^ (a chamber with a 3.5‐cm diameter) (Figure 4D). Seven serially connected SCD3s, fully charged with 1 × 10^8^ SCs in each cell pool, could produce a total output voltage of at least 2.8 V and power a commercial LED with an input threshold of 2.3 V with uniform illumination (Movie S1, Supporting Information). This confirmed that the SCD technology was indeed scalable. Furthermore, from a safety point of view, previous studies of battery‐powered electrogenetic interfaces, stimulating cultured human cells at 4.5 V, did not produce any detectable amount of toxic chlorine gas (< 0.01 ppm) and had no negative impact on the viability of the cells,^[^ ^36^ ^]^ as was the case for the SCD3 devices after 30 min of illumination.

## 3 Discussion and Conclusion

Current renewable energy sources, such as solar energy, wind energy or hydroelectric power, typically have high installation costs and require significant maintenance and specialized equipment.^[^ ^37^ ^]^ Technologies for harvesting electrical energy from biological sources remain in their infancy,^[^ ^21^, ^38^ ^]^ although some abiotic power‐generating systems with principles analogous to a concentration‐difference battery, such as in the electric organ of an electric eel, have been reported.^[^ ^22^, ^23^, ^24^, ^25^ ^]^ Here, we adopted an eel‐mimicking strategy to construct miniaturized devices containing mammalian photovoltaic cells capable of generating sustained ion concentration gradients that could be used to generate electricity. These devices not only enabled light‐dependent, reversible charge‐discharge cycles and sustained electrical potential generation in open‐circuit testing (0.4 V from a single device) for more than 12 h, but were also modular, affording scalable electrical power (≈0.1 mW cm^−2^).

Light‐activated ion channels and pumps have been extensively studied in recent years,^[^ ^39^, ^40^, ^41^, ^42^ ^]^ and have been applied to the regulation of neuronal activities for mechanistic studies^[^ ^27^, ^29^, ^31^, ^43^, ^44^, ^45^ ^]^ and for the modulation of gene expression in synthetic biology‐inspired cell therapies.^[^ ^39^, ^40^, ^41^, ^42^ ^]^ In the present work, we exploited their ability to modulate extracellular ion levels to develop a cell‐based light‐tunable device for generating sustained cation concentration gradients, and thereby, generating electricity. A key feature of this study was the empirical testing of many combinations of ion channels and pumps to optimize current generation. The monoclonal cell line SC, stably expressing hChR2(H134R), C1V1(t/t), and eArch3.0, was identified as a promising candidate for a cell‐based photovoltaic battery (“solar cell”) capable of generating electricity from ion gradients. The operating wavelength range of the SC containing these elements was extended from the bandwidth of the individual channels (50–80 nm) to ≈440–600 nm, which would enable energy to be harvested from the broad spectrum of natural sunlight.^[^ ^32^ ^]^ Particularly, the proof‐of‐concept results with SCD3 showed that the optimized combination of bio‐engineered photosensitive constructions and building‐up of cell resistance to ion strength changes could dramatically improve the solar energy collection in our photovoltaic system. We chose to use simulated sunlight (white light) to obtain precise illumination control for consistency throughout the experimental work; however, natural sunlight with a similar power density and spectrum should serve as a sustainable energy source in the future.

In contrast to the electron‐transfer‐chain‐based systems previously developed for power generation in biological systems,^[^ ^16^, ^17^, ^18^ ^]^ our photovoltaic system does not require intensive electrochemical reactions and hence, does not produce high concentrations of superoxide ions or cause excessive heating.^[^ ^27^, ^28^ ^]^ Thus, these engineered mammalian cells work in an environment with greater biocompatibility, which should extend the longevity of the cell‐based device. Previously, a retinal‐cell‐based fuel battery has been reported;^[^ ^20^ ^]^ however, to our knowledge, our SCD device is the first mammalian system for power generation that is entirely powered by solar energy. Such an implantable energy‐harvesting system has the potential for sustainable long‐term operation. Importantly, its voltage output reaches the same level as that of metabolic fuel cells, which convert energy from supplementary blood glucose, and is sufficient to trigger opto‐,^[^ ^30^ ^]^ or electro‐genetic control of insulin release.^[^ ^5^, ^6^ ^]^ Thus, we believe our photovoltaic design can provide the basis for energy‐efficient continuous power sources for implantable or wearable biosensors and trigger‐responsive opto‐ or electro‐genetic interfaces.

## 4 Experimental Section

### Plasmid Construction

Comprehensive design and construction details for all expression vectors are listed in Table S1 (Supporting Information). Some of the plasmids were constructed using Gibson assembly kits (New England Biolabs, Beverly, MA; cat. no. E2611L).^[^ ^46^ ^]^ All new constructs were confirmed by Sanger sequencing (Microsynth AG). p‐mCherry‐C1‐iChR2++ (Addgene plasmid #98172) was provided by Peter Hegemann from Humboldt–Universtät zu Berlin. pcDNA3.1_PsChR2_EYFP (Addgene plasmid #69057) was provided by John Spudich from McGovern Medical School. AAV‐CAG‐hChR2‐H134R‐tdTomato (Addgene plasmid #28017) was provided by Karel Svoboda. pAAV‐Ef1a‐DIO‐C1V1(t/t)‐TS‐EYFP (Addgene plasmid #35497), pLenti‐CaMKIIa‐eMac3.0‐EYFP (Addgene plasmid #35515), pAAV‐CaMKIIa‐eArch3.0‐EYFP (Addgene plasmid #35516), and pAAV‐Ef1a‐DIO eNpHR3.0‐EYFP (Addgene plasmid #26966) were provided by Karl Deisseroth from Alle Institute. pAAV‐EF1a‐DIO‐iChloC‐2A‐dsRed (Addgene plasmid #70762) was provided by Thomas Oertner from the Center for Molecular Neurobiology Hamburg.

### Cell Culture and Transfection

Human embryonic kidney cells (HEK‐293, ATCC: CRL‐11268) were cultivated in DMEM (Thermo Fisher Scientific, Waltham, MA; cat. no. 52100‐39), supplemented with 10% fetal bovine serum (FBS; Sigma–Aldrich, Burlington, MA; cat. no. F7524; lot no. 022M3395) and 1% (v/v) streptomycin/penicillin (Biowest, Nuaillé, France; cat. no. L0022), and grown at 37 °C under a humidified atmosphere containing 5% CO_2_. All cell lines were transfected using an optimized polyethyleneimine (PEI)‐based protocol.^[^ ^46^ ^]^ For cell culture experiments, 5 × 10^6^ cells per 10 cm dish were seeded 18 h before transfection and incubated for 6 h with 1 mL of a 3:1 PEI/DNA mixture (w/w) (PEI: molecular weight 40000, stock solution 1 mg mL^−1^ in ddH_2_O; Polysciences, Warrington, PA; cat. no. 24765), containing 15 µg total DNA in serum‐ and antibiotic‐free DMEM. Cell viability was quantified using a cell counting kit‐8 (Sigma–Aldrich; cat. no. 96992) according to the manufacturer's instructions, and the pH of culture supernatants was profiled using a SevenExcellence S500 pH meter (Mettler Toledo, Zurich, Switzerland). Chloride ion concentration was measured with an electrolyte analyzer (K‐lite6D Electrolyte Analyzer, Meizhou Cornley High‐Tech Co., Ltd.).

### Generation and Validation of Stable Cell Lines

Stable cell lines were generated by co‐transfection of HEK‐293 cells with a constitutive expression vector for the Sleeping Beauty transposase SB100X (pTS395, P_hCMV_‐SB100X‐pA),^[^ ^47^ ^]^ and corresponding SB100X‐specific transposons for the expression of light‐sensitive ion channels or pumps (pXS630, pXS631, pXS632, pXS633, pXS720, pXS721; Table S1, Supporting Information) at a plasmid ratio of 1:20 (w/w). At 24 h after transfection, the cells were transferred to a medium containing appropriate antibiotics and cultured for a 2‐week selection round. Cells were randomly selected from the cell populations that survived each selection round (Table 1) and expanded. All the selected clones were subjected to a continuous 2‐week sensitizing program consisting of alternating cycles of a 30‐min exposure to white light at 3 mW cm^−2^, followed by 2 h in the dark. To identify the best‐performing stably‐transgenic monoclonal cell lines for each of the 11 genetic configurations, 10 cell clones with the highest fluorescence, as a proxy for co‐expressed light‐sensitive membrane proteins, were subjected to potential difference measurements in the designed device (SCD). Engineered cells transiently or stably expressing multiple photosensitive ion channels were exposed to white light in SCD1 for voltage production evaluation (Table 1). All assays were performed using a light intensity of 3 mW cm^−2^ and a cell density of 10^7^ cells mL^−1^ (0.5 mL in the insert).

### Cl_2_ Gas Measurement

Chlorine gas, dissolved in a culture medium, was analyzed using test strips (DPD‐1, cat. no. 486637, ITS Europe), which were quantified by a photometer (eXact EZ Photometer, cat. no. 486205, ITS Europe).

### Design and Fabrication of the SCD

As shown in Figure 1, the SCD1 was constructed from VWR 12‐well tissue culture plate inserts with a pore size of 0.1 µm (VWR International, Radnor, PA; cat. no. 734‐2729), which were coated at the bottom exterior with an ion‐selective Nafion 117 solution (Sigma–Aldrich, cat. no. 70160) and mounted on a 12‐well cell culture plate (Corning, Glendale, AZ; cat. no. 353225). Cells were seeded onto the insert with two hanging platinum electrodes placed on opposite sides of the ion‐selective coating (Figure 1B). The SCD2 design consisted of seven stacked components (Figure 2B), including a transparent PET sealing film (Sigma‐Aldrich, cat. no. GF25214475), two polylactic (PLA) chambers, two electrodes, and an ion exchange film (Nafion perfluorinated membrane, Sigma Aldrich, cat. no. 915270). The PLA chambers were fabricated using a Prusa MINI + 3D printer (Prusa, Czech Republic). The reinforcement ring was attached to the cell culture chamber using epoxy resin (Epoxy Technology Inc., Billerica, MA; cat. no. 301‐2), and the adhesive was allowed to set overnight at 37 °C, followed by a 2‐day wash in 500 ml of ddH_2_O and UV sterilization. For animal experiments, a porous polycarbonate membrane (Whatman Nuclepore track‐etched membranes, Sigma‐Aldrich, cat. no. WHA111156) was used on the cell pool.

### Electrical Characterization of SCD

*V_OC_* and short‐circuit current were measured and quantified using a CHI 760 potentiostat (CH Instruments Inc., Austin, TX). Real‐time current and voltage measurements were recorded using a Keithley 2636B source measurement unit (Tektronix, Beaverton, OR). Resistor devices were connected to the SCD with a platinum wire for characterization of the charge–discharge process (200 KΩ, YAGEO, MFR‐12FTF52‐2 M), and for reversibility characterization (10 MΩ, YAGEO, MFR‐12FTF52‐10 M).

Power density (PD) analysis was performed by linear sweep voltammetry,^[^ ^48^ ^]^ using a platinum anode as the working electrode, and with the counter and reference electrodes shorted to a platinum wire cathode for electrical measurement. *V_OC_* and PD were calculated from the anode surface area. The power was calculated according to Equation (1):

(1) P=V×I,

where *P* is the power (W); *V* is the voltage (V); and *I* is the current (A).

The efficiency of the solar cell, denoted as η, is calculated using Equation (2):

(2) $$\eta =\left(P_{\mathrm{out}}/P_{\mathrm{in}}\right)\times 100\%$$

where *P*_out_ is the output power density and *P*_in_ is the input light power density. From the results, the output power density is 0.08 µW cm^−^^2^, the light intensity is 3 mW cm^−^^2^, which is equivalent to 3000 µW cm^−^^2^. Consequently, η = (0.08/3000) × 100%, which results in an efficiency of ≈0.0027%.

For LED illumination, an LED (Roithner Lasertechnik, cat. no. LED565‐33u) was connected to the SCD with a platinum wire.

### Animal Experiments

Animal experiments were performed according to a protocol (Protocol ID: AP#24‐096‐XMQ) approved by the Institutional Animal Care and Use Committee (IACUC) of Westlake University (People's Republic of China), and in accordance with the Animal Care Guidelines of the Ministry of Science and Technology of the People's Republic of China. 10‐ to 12‐week‐old male wild‐type C57BL/6JRJ mice (average weight ≈25–30 g) were housed in a temperature (21 ± 2 °C) and humidity (55 ± 10%) controlled room on a 12‐h inverse daylight cycle with free access to standard chow and drinking water, before random assignment to different experimental groups. The dorsal region of the mice was shaved. Animals were anesthetized with 4% isoflurane before implantation and maintained under 2% inhalational isoflurane anesthesia during surgery. An SCD2 loaded with 1 × 10^7^ SCs mL^−1^ was implanted subcutaneously on the dorsal side; the wound opening was sutured and closed with a surgical clip (Clay Adams AUTOCLIP wound clip; Becton Dickinson Primary Care Diagnostics, cat. no. 427 631) and connected to two platinum electrodes. The experimental group was exposed to light (light denity: 3 mW cm^−2^) while the control group was kept in the dark before the output voltage levels were measured.

### Statistical Analysis

Statistical significance of differences between groups was evaluated with a two‐tailed, unpaired Student's t‐test using GraphPad Prism 9 (v.9.5.1, GraphPad Software) or Microsoft Excel (v.16.80, Microsoft). One‐way ANOVA was used to compare differences among more than two groups. Differences were considered statistically significant at P < 0.05. All data presented were obtained in independently repeated experiments, as indicated in the figure legends.

## Conflict of Interest

The authors declare no conflict of interest.

## Author Contributions

S.X. and Z.L. contributed equally to this work. S.X. and M.F. designed the project. S.X., Z.L., D.M., and P.G.R. conducted device fabrication and electrical characterization as well as the in vitro experiments. S.X., Z.L., D.M., P.G.R., M.X., and M.F. designed the experiments and analyzed the results. S.X. performed the animal experiments. S.X., Z.L., P.G.R., M.X., and M.F. prepared the manuscript.

## Supporting information



Supplemental Movie 1

Solar Cells‐Supporting Information

## Data Availability

The data that support the findings of this study are available from the corresponding author upon reasonable request.
