# Supplementary material for: Implantable Light‐Powered Human Designer Cells for Electrical Energy Generation
Source: Adv Mater. 2025 Sep 3;37(45):e02618. doi: 10.1002/adma.202502618 (PMC12617042; doi:10.1002/adma.202502618)
Supplement: Supplementary file 2 — Solar Cells‐Supporting Information [file ADMA-37-e02618-s002.docx]

**Supplementary Information**

Implantable Light-Powered Human Designer Cells for Electrical Energy Generation

*Shuai Xue^#^, Zhihua Lin^#^, Debasis Maity, Preetam Guha Ray, Mingqi Xie, Martin Fussenegger**

*Corresponding author e-mail: fussenegger@bsse.ethz.ch

S. Xue, Z. Lin, D. Maity, P. Guha Ray, M. Fussenegger

Department of Biosystems Science and Engineering

ETH Zürich

Klingelbergstrasse 48, Basel CH-4056, Switzerland

E-mail: fussenegger@bsse.ethz.ch

S. Xue, M. Xie

Westlake Laboratory of Life Sciences and Biomedicine

Hangzhou, Zhejiang 310024, China

M. Fussenegger

Faculty of Science

University of Basel

Klingelbergstrasse 48, Basel CH-4056, Switzerland

**Table of Contents**

Figure S1-2

Table S1

References

**
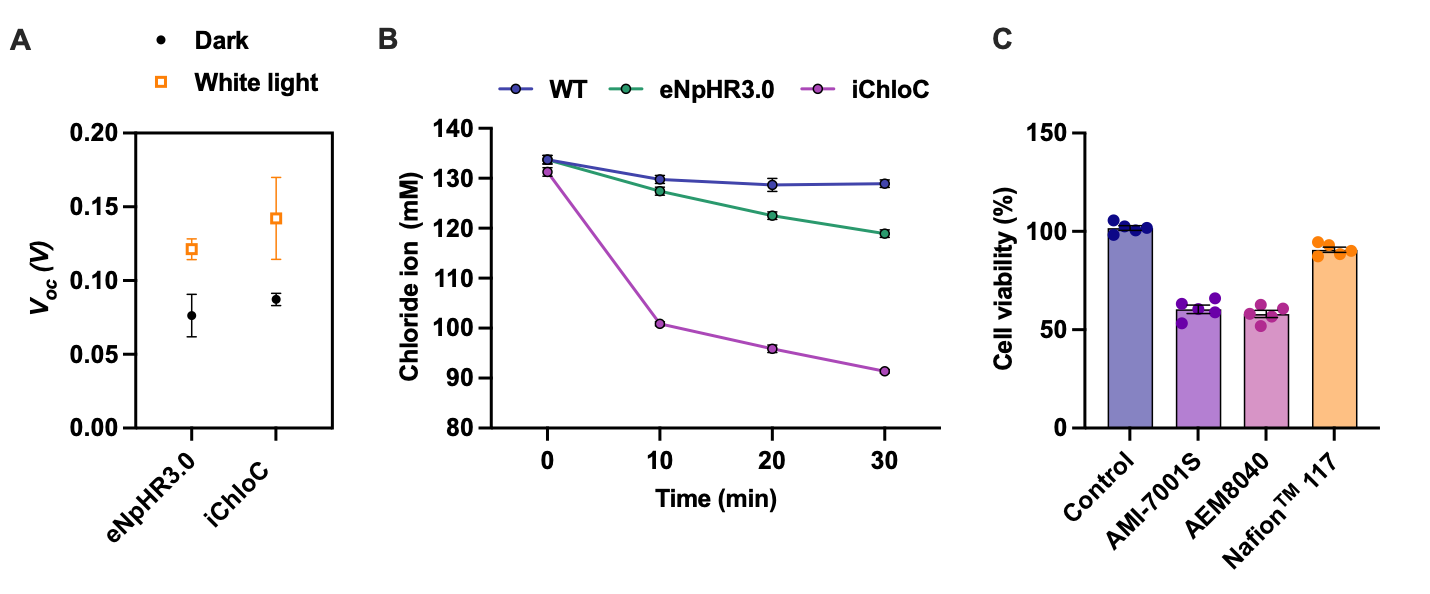
**

**Figure S1.** SCD1 with anion exchange membrane (AEM) and stable designer cells engineered with a chloride-channel or pump. (A) Voltage generation immediately after illumination (cell density, 1 × 10^7^ cells/mL; light intensity, 3 mW/cm^2^, 30 min; AEM, AMI-7001S). (B) Concentration changes of chloride ions immediately after illumination (cell density, 1 × 10^7^ cells/mL; light, 3 mW/cm^2^, 30 min). (C) Viability of stable designer cells expressing iChloC after a 24-hour coculture with different ion exchange membranes. Data in (A, B, C) have been presented as mean ± SEM, (A, B) n = 3 and (C) n = 6.


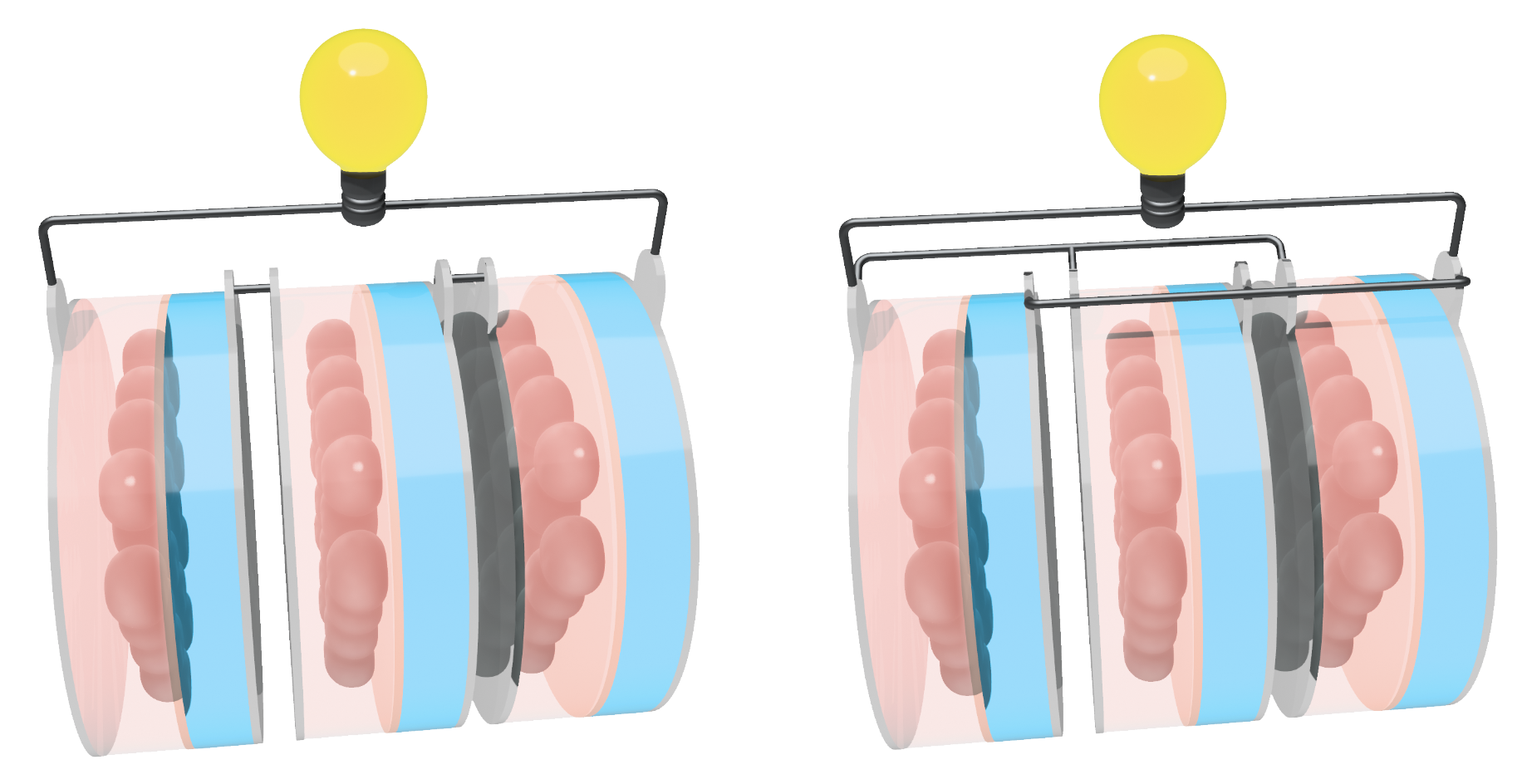


**Figure S2. Schemes of series and parallel linkage of three SCD2s.**

**Table S1. Plasmids designed and used in this study**

| **Plasmid** | **Information and Design** | **Reference** |
| --- | --- | --- |
| pcDNA3.1(+) | Mammalian expression vector (P_hCMV_-MCS-pA). | Life Technologies, Carlsbad, CA |
| pTS395 | Constitutive mammalian SB100X expression vector (P_hCMV_-SB100X-pA). | (Haellman et al., 2021)^[1]^ |
| pJH1096 | SB100X-specific transposon containing a P_DART_-driven expression unit for NanoLuc and mINS and a constitutive ZeoR expression unit (ITR-P_DART_-NanoLuc-P2A-mINS-pA:P_mPGK_-ZeoR-pA-ITR). | (Huang et al., 2023)^[2]^ |
| pXS101 | SB100X-specific transposon containing a constitutive expression unit for CREB1-TetR, mCherry and PuroR (ITR-P_hCMV_-CREB1-TetR-2A-mCherry-2A-PuroR-pA-ITR). | (Xue et al., 2017)^[3]^ |
| pMX251 | SB100X-specific transposon containing a constitutive expression unit for *Cacna2d1* and *Cacnb3* and a constitutive expression unit for dTomato and BlastR (ITR-P_hEF1α_-Cacna2d1-P2A-Cacnb3-pA:P_RPBSA_-dTomato-P2A-BlastR-pA). | (Xie et al., 2016)^[4]^ |
| pMX252 | SB100X-specific transposon containing a constitutive *Cacna1d* expression unit and a constitutive expression unit for BFP and PuroR (ITR-P_hEF1α_-Cacna1d-pA: P_RPBSA_-BFP-P2A-PuroR-pA-ITR). | (Xie et al., 2016)^[4]^ |
| pXS610 | SB100X-specific transposon containing a constitutive expression unit for mCherry-tagged iChR2^++^ and a constitutive PuroR expression unit (ITR-P_hCMV_-mCherry-C1-iChR2^++^-pA:P_mPGK_-PuroR-pA-ITR).  mCherry-C1-iChR2^++^ was PCR-amplified from p-mCherry-C1-iChR2++ (Addgene plasmid #98172) using oligonucleotides 5’- GAACCGTCAGATCCGCTAGCCACCATGGATTATGGGGGAGCACTG-3’ and 5’- TACCCGGTAGAATTAGGATCCTTACTTGTACAGCTCGTCCATGCC-3’, the Sleeping Beauty transposon backbone was PCR-amplified from pXS101 using oligonucleotides 5’- ATCTCCGGGCCTTTCGACCTGCAGCCCAAGCTTACCATGGCCACCGAGTACAAG-3’ and 5’- TAATCCATGGTGGCTAGCGGATCTGACGGTTCACTAAACCAGCTCTGCTTATATAG-3’, P_mPGK_ was PCR-amplified from pJH1096 using oligonucleotides 5’-CTGTACAAGTAAGGATCCTTCTACCGGGTAGGGGAGGCGCTTTTCCC-3’and 5’- GGTAAGCTTGGGCTGCAGGTCGAAAGGCCCGGAGAT-3’. All fragments were assembled through Gibson cloning. | This work |
| pXS612 | SB100X-specific transposon containing a constitutive expression unit for EYFP-tagged PsChR2 and a constitutive BlastR expression unit (ITR-P_hCMV_-PsChR2_EYFP-pA:P_mPGK_-BlastR-pA-ITR).  P_hCMV_-PsChR2_EYFP was PCR-amplified from pcDNA3.1_PsChR2_EYFP (Addgene plasmid #69057) using oligonucleotides 5’-CAGTTTGGTTAATTAGCTAGACGTTACATAACTTACGGTAAATGGCCCGCCTGGCTGA-3’ and 5’-TACCCGGTAGAATTAGGATCCTTACTTGTACAGCTCGTCCATGCC-3’, the Sleeping Beauty transposon backbone was PCR-amplified from pMX251 using oligonucleotides 5’- GTTATGTAACGTCTAGCTAATTAACCAAACTGG-3’ and 5’-TAATCCATGGTGGCTAGCGGATCTGACGGTTCACTAAACCAGCTCTGCTTATATAG-3’, P_mPGK_ was PCR-amplified from pJH1096 using oligonucleotides 5’-TGTACAAGTAAGGATCCTAATTCTACCGGGTAGGGGAGGCGCTTTTCCC-3’and 5’-ATCTCCGGGCCTTTCGACCTGCAGCCCAAGCTTACC-3’. All fragments were assembled through Gibson cloning. | This work |
| pXS630 | SB100X-specific transposon containing a constitutive expression unit for tdTomato-tagged hChR2(H134R) and a constitutive PuroR expression unit (ITR-P_hEF1α_-hChR2(H134R)-tdTomato-pA:P_mPGK_-PuroR-pA-ITR).  hChR2(H134R)-tdTomato was PCR-amplified from AAV-CAG-hChR2-H134R-tdTomato (Addgene plasmid #28017) using oligonucleotides 5’- TTCGAATTCGATCTAGCGCCACCATGGACTATGGCGGCGCTTTGTCT-3’ and 5’- GCGCCTCCCCTACCCGGTAGAAGGATCCTTACTTGTACAGCTCGTCCATGCCGTACAG-3’, the Sleeping Beauty transposon backbone was PCR-amplified from pMX252 using oligonucleotides 5’-GGTGGCGCTAGATCGAATTCGAATCGACTAGCTCACGACACCTGAAATGGAAG-3’ and 5’- ATCTCCGGGCCTTTCGACCTGCAGCCCAAGCTTACCATGGCCACCGAGTACAAG-3’, P_mPGK_ was PCR-amplified from pJH1096 using oligonucleotides 5’- TAAGGATCCTTCTACCGGGTAGGGGAGGCGCTTTTCCCAAGGC-3’and 5’- GGTAAGCTTGGGCTGCAGGTCGAAAGGCCCGGAGAT-3’. All fragments were assembled through Gibson cloning. | This work |
| pXS631 | SB100X-specific transposon containing a constitutive expression unit for EYFP-tagged C1V1(t/t) and a constitutive BlastR expression unit (ITR-P_hEF1α_-C1V1(t/t)-EYFP-pA:P_mPGK_-BlastR-pA-ITR).  C1V1(t/t)-TS-EYFP was PCR-amplified from pAAV-EF1a-DIO-C1V1(t/t)-TS-EYFP (Addgene plasmid #35497) using oligonucleotides 5’-CTTCCATTTCAGGTGTCGTGAGCTAGCCACCATGTCGCGGAGGCCATGGCTTCTTGCC-3’ and 5’- TACCCGGTAGAATTAGGATCCTTACTTGTACAGCTCGTCCATGCC-3’, the Sleeping Beauty transposon backbone was PCR-amplified from pMX251 using oligonucleotides 5’-GGTGGCGCTAGATCGAATTCGAATCGACTAGCTCACGACACCTGAAATGGAAG-3’ and 5’- CCGGGCCTTTCGACCTGCAGCCCAAGCTTACCATGGCCAAGCCTTTGTCTCAAGAAGAATCC-3’, P_mPGK_ was PCR-amplified from pJH1096 using oligonucleotides 5’- TAAGGATCCTTCTACCGGGTAGGGGAGGCGCTTTTCCCAAGGC-3’and 5’- GGTAAGCTTGGGCTGCAGGTCGAAAGGCCCGGAGAT-3’. All fragments were assembled through Gibson cloning. | This work |
| pXS632 | SB100X-specific transposon containing a constitutive expression unit for EYFP-tagged eNpHR3.0 and a constitutive ZeoR expression unit (ITR-P_hEF1α_-eNpHR3.0-EYFP-pA:P_mPGK_-ZeoR-pA-ITR).  eNpHR 3.0-EYFP was PCR-amplified from pAAV-EF1α-DIO-eNpHR3.0-EYFP (Addgene plasmid #26966) using oligonucleotides 5’-CTTCCATTTCAGGTGTCGTGAGCTAGCGCCACCATGACAGAGACCCTGCCTCCCGTG -3’ and 5’-TACCCGGTAGAATTAGGATCCTTACTTGTACAGCTCGTCCATGCC-3’, the Sleeping Beauty transposon backbone was PCR-amplified from pJH1096 using oligonucleotides TAAGGATCCTTCTACCGGGTAGGGGAGGCGCTTTTCCCAAGGC-3’ and 5’- CCGGGCCTTTCGACCTGCAGCCCAAGCTTACCATGGCCAAGCCTTTGTCTCAAGAAGAATCC-3’. All fragments were assembled through Gibson cloning. | This work |
| pXS633 | SB100X-specific transposon containing a constitutive expression unit for iChloC and dsRed and a constitutive ZeoR expression unit (ITR-P_hEF1α_-iChloC-2A-dsRed-pA:P_mPGK_-ZeoR-pA-ITR).  ChloC-2A-dsRed was PCR-amplified from pAAV-EF1α-DIO-iChloC-2A-dsRed (Addgene plasmid #70762) using oligonucleotides 5’- GGTGTCGTGAGCTAGCCACCATGGATTATGGAGGCGCCCTGAGT-3’ and 5’- GGATCCTTACTTGTACATTACAGGAACAGGTGGTGGCGGCCCTCGGAGCG-3’, the Sleeping Beauty transposon backbone was PCR-amplified from pJH1096 using oligonucleotides TAAGGATCCTTCTACCGGGTAGGGGAGGCGCTTTTCCCAAGGC-3’ and 5’- CCGGGCCTTTCGACCTGCAGCCCAAGCTTACCATGGCCAAGCCTTTGTCTCAAGAAGAATCC-3’. All fragments were assembled through Gibson cloning. | This work |
| pXS720 | SB100X-specific transposon containing a constitutive expression unit for EYFP-tagged eArch3.0 and a constitutive ZeoR expression unit (ITR-P_hEF1α_-eArch3.0-EYFP-pA:P_mPGK_-ZeoR-pA-ITR).  eArch3.0-EYFP was PCR-amplified from pAAV-CaMKIIa-eArch3.0-EYFP (Addgene plasmid #35516) using oligonucleotides 5’-CCATTTCAGGTGTCGTGAGCTAGCCACCATGGACCCCATCGCTCTG-3’ and 5’-GCGTATTTAAATTAATTAATTACTTGTACAGCTCGTCCATGCC-3’, the Sleeping Beauty transposon backbone was PCR-amplified from pXS632 using oligonucleotides 5’- TGTACAAGTAAGGATCCTTCTACCGGGTAGGGGAGGCGCTTTTCCC-3’ and 5’-GGTGGCTAGCTCACGACACCTGAAATGGAAG-3’, pA was PCR-amplified from pJH1096 using oligonucleotides 5’- TAATTAATTAATTTAAATACGCGTTTAAACAGATCC-3’and 5’- GGTAGAAGGATCCTTACTTGTACATAAGATACATTGATGAGTTTGG-3’. All fragments were assembled through Gibson cloning. | This work |
| pXS721 | SB100X-specific transposon containing a constitutive expression unit for EYFP-tagged eMac3.0 and a constitutive ZeoR expression unit (ITR-P_hEF1α_-eMac3.0-EGFP-pA:P_mPGK_-ZeoR-pA-ITR)  eMac3.0-EGFP was PCR-amplified from pLenti-CaMKIIa-eMac3.0-EYFP (Addgene plasmid #35515) using oligonucleotides 5’- CAGGTGTCGTGAGCTAGCCACCATGATCGTGGACCAGTTCGAGGAG-3’ and 5’- GCGTATTTAAATTAATTAATTACTTGTACAGCTCGTCCATGCC-3’, the Sleeping Beauty transposon backbone was PCR-amplified from pXS632 using oligonucleotides 5’- TGTACAAGTAAGGATCCTTCTACCGGGTAGGGGAGGCGCTTTTCCC-3’ and 5’-GGTGGCTAGCTCACGACACCTGAAATGGAAG-3’, pA was PCR-amplified from pJH1096 using oligonucleotides 5’- TAATTAATTAATTTAAATACGCGTTTAAACAGATCC-3’and 5’- GGTAGAAGGATCCTTACTTGTACATAAGATACATTGATGAGTTTGG-3’. All fragments were assembled through Gibson cloning. | This work |

**Oligonucleotides:** Restriction endonuclease-specific sites are underlined, annealing base pairs are indicated in capital letters.

**Abbreviations**:

**BFP**, Blue fluorescent protein; **BlastR**, gene conferring blasticidin resistance; **C1V1(t/t)**, chimeric opsin variant of C1V1(E122T/E162T) for fast and potent optical excitation at red-shifted wavelengths;^[5]^ **Cacna1d**, α1-subunit of rat Cav1.3 (NCBI Gene ID: 29716); **Cacnb3**, β3-subunit of rat Cav1.3 (NCBI Gene ID: 25297); **Cacna2d1**, α2δ-subunit of rat Cav1.3 (NCBI Gene ID: 25399); **CREB1**, cyclic AMP-responsive element binding protein1; **dsRed**, a red fluorescent protein; **dTomato**, destabilized red fluorescent protein variant; **eArch3.0**, *Halorubrum sodomense*-derived outward proton pump;^[6]^ **EGFP**, enhanced green fluorescent protein; **eMac3.0**, *Leptosphaeria maculans*-derived outward proton pumps;^[6]^ **eNpHR3.0**, third-generation *Natromonas pharaonis* halorhodopsin;^[7]^ **EYFP**, enhanced yellow fluorescent protein; **hChR2****(H134R)**, channelrhodopsin-2 mutant with a H134R amino-acid substitution;^[5]^ **iChloC**, chloride-conducting channelrhodopsin;^[8]^ **iChR2^++^**, artificial anion conducting channelrhodopsin;^[9]^ **ITR**, inverted terminal repeats of SB100X; **mCherry**, mushroom coral red fluorescent protein; **MCS**, multiple cloning site; **mINS**, modified furin-cleavable insulin variant;^[10]^ **NanoLuc**, secreted *Oplophorus gracilirostris* luciferase containing a IgK-derived signal peptide; **PCR**, polymerase chain reaction; **P_CAG_**, synthetic mammalian chimeric promoter containing elements derived from the cytomegalovirus early enhancer (C), chicken β-actin promoter (A) and the splice acceptor of the rabbit beta-globin gene (G); **P_DART_**, synthetic reactive oxygen species (ROS)-responsive mammalian promoter;^[2]^ **P_hCMV_**, human cytomegalovirus immediate promoter; **P_hEF1α_**, human elongation factor 1α promoter; **P_mPGK_**, murine phosphoglycerate kinase gene promoter; **P_RPBSA_**, constitutive synthetic mammalian promoter; **P2A**, porcine teschovirus-derived ribosome skipping sequence optimized for bicistronic expression in mammalian cells; **pA**, polyadenylation signal; **PsChR2**, high-efficiency blue-shifted cation channelrhodopsin;^[11]^ **PuroR**, gene conferring puromycin resistance; **SB100X**, optimized Sleeping Beauty transposase;^[12]^ **TetR**, *Escherichia coli* Tn10-derived tetracycline-dependent repressor of the tetracycline resistance gene; **ZeoR**, gene conferring zeocin resistance.

**References:**

[1] V. Haellman, T. Strittmatter, A. Bertschi, P. Stucheli, M. Fussenegger, Metab. Eng. **2021**, 66, 41.

[2] J. B. Huang, S. Xue, P. Buchmann, A. P. Teixeira, M. Fussenegger, Nat. Metab. **2023**, 5, 1395.

[3] S. Xue, J. L. Yin, J. W. Shao, Y. H. Yu, L. F. Yang, Y. D. Wang, M. Q. Xie, M. Fussenegger, H. F. Ye, Mol. Ther. **2017**, 25, 443.

[4] M. Xie, H. Ye, H. Wang, G. Charpin-El Hamri, C. Lormeau, P. Saxena, J. Stelling, M. Fussenegger, Science **2016**, 354, 1296.

[5] T. Y. Mao, D. Kusefoglu, B. M. Hooks, D. Huber, L. Petreanu, K. Svoboda, Neuron **2011**, 72, 111.

[6] J. Mattis, K. M. Tye, E. A. Ferenczi, C. Ramakrishnan, D. J. O'Shea, R. Prakash, L. A. Gunaydin, M. Hyun, L. E. Fenno, V. Gradinaru, O. Yizhar, K. Deisseroth, Nat. Methods **2012**, 9, 159.

[7] V. Gradinaru, F. Zhang, C. Ramakrishnan, J. Mattis, R. Prakash, I. Diester, I. Goshen, K. R. Thompson, K. Deisseroth, Cell **2010**, 141, 154.

[8] J. Wietek, R. Beltramo, M. Scanziani, P. Hegemann, T. G. Oertner, J. S. Wiegert, Sci. Rep. **2015**, 5, 14807.

[9] J. Wietek, S. Rodriguez-Rozada, J. Tutas, F. Tenedini, C. Grimm, T. G. Oertner, P. Soba, P. Hegemann, J. S. Wiegert, Sci. Rep. **2017**, 7.

[10] C. W. Hay, K. Docherty, J. Mol. Endocrinol. **2003**, 31, 597.

[11] E. G. Govorunova, O. A. Sineshchekov, H. Li, R. Janz, J. L. Spudich, J. Biol. Chem. **2013**, 288, 29911.

[12] L. Mátés, M. K. Chuah, E. Belay, B. Jerchow, N. Manoj, A. Acosta-Sanchez, D. P. Grzela, A. Schmitt, K. Becker, J. Matrai, L. Ma, E. Samara-Kuko, C. Gysemans, D. Pryputniewicz, C. Miskey, B. Fletcher, T. VandenDriessche, Z. Ivics, Z. Izsvák, Nat. Genet. **2009**, 41, 753.
